# Improving work for the body – a participatory ergonomic intervention aiming at reducing physical exertion and musculoskeletal pain among childcare workers (the TOY-project): study protocol for a wait-list cluster-randomized controlled trial

**DOI:** 10.1186/s13063-018-2788-z

**Published:** 2018-07-31

**Authors:** Charlotte Diana Nørregaard Rasmussen, Peter Rasmus Hendriksen, Malene Jagd Svendsen, Dorte Ekner, Klaus Hansen, Ole Henning Sørensen, Susanne Wulff Svendsen, Allard J. van der Beek, Andreas Holtermann

**Affiliations:** 1 National Research Centre for the Working Environment, Lersø Parkallé 105, 2100 Copenhagen, Denmark; 2 Center for Industrial Production, Aalborg University Copenhagen, A. C. Meyers Vænge 15, 2450 Copenhagen, Denmark; 3 Danish Ramazzini Centre, Department of Occupational Medicine, Regional Hospital West Jutland—University Research Clinic, Herning, Denmark; 4 Department of Public and Occupational Health, Amsterdam Public Health Research Institute, VU University Medical Center, Van der Boechorststraat 7, 1081 BT Amsterdam, The Netherlands; 5 Department of Sports Science and Clinical Biomechanics, University of Southern Denmark, Campusvej 55, 5230 Odense M, Denmark

**Keywords:** Musculoskeletal disorders, Participatory ergonomics, Workplace intervention

## Abstract

**Background:**

The prevalence of musculoskeletal pain (MSP) is persistently high throughout the world. Work-related factors such as high physical workload (lifting, bending and twisting of the back) are considered to be among the main causes of MSP. Work in childcare includes the need to lift, carry, and support children in a range of activities, requiring several demanding postures and movements, such as bending forward and twisting of the back and sitting on the floor. Participatory ergonomics may represent a solution for decreasing the physical workload to reduce MSP. We present the protocol of a study aiming to evaluate the effect and process of a participatory ergonomics intervention designed to reduce physical exertion during work and MSP (including MSP interfering with work) among childcare workers.

**Methods/design:**

This study will use a two-arm cluster-randomized design employing a wait-list control, with childcare institutions forming the clusters. Three workshops will be conducted during the 4-month intervention period. Participants will identify risk factors for strenuous work and MSP, develop solutions for reducing the identified risk factors, and implement them in their team. An ergonomic consultant will guide the process. The data collection will consist of questionnaires and objective measures of heart rate and physical activity, observations of physical workload, and information on sickness absence based on company records. Primary outcomes are physical exertion during work and MSP (including pain-related work interference) measured at 4 months. Secondary outcomes measured at 4 months are sickness absence due to MSP; objectively measured occupational physical activity and heart rate; and self-reported self-efficacy, employee involvement, and need for recovery. Alongside the trial, a process evaluation and an economic evaluation will be conducted.

**Discussion:**

The study will evaluate the effect and process of a participatory ergonomics intervention to reduce physical exertion at work and MSP among childcare workers. By performing a cluster-randomized controlled trial with an effect evaluation based on both objective and self-reported measures with the addition of a process evaluation and economic evaluation, this study will contribute to the evidence for prevention of MSP among a less studied occupational group. Results are expected in 2018–2019.

**Trial registration:**

ISRCTN, ISRCTN10928313. Registered on 11 January 2017.

**Electronic supplementary material:**

The online version of this article (10.1186/s13063-018-2788-z) contains supplementary material, which is available to authorized users.

## Background

The prevalence of and burden from musculoskeletal pain (MSP) is high throughout the world and is estimated to cause 21% of the total years lived with disability [1]. Causes of MSP are multifactorial [2]. However, work-related factors are considered to be among the main causes of MSP. These factors particularly involve high physical workload (lifting, bending and twisting of back) and work-related psychosocial factors (e.g., stress, social support, job satisfaction and job control) [3]. The workplace is therefore an important setting for implementation of preventive measures for MSP.

The physical workload in childcare includes the need to lift, carry, and support children in a range of activities, requiring several demanding body postures and movements, such as bending forward and twisting of the back and sitting on the floor [4]. Danish childcare workers report a high physical workload, high physical exertion during work, a high prevalence of MSP, and a high prevalence of sickness absence [5]. Thus, there is a need for effective and feasible interventions for reducing the high physical exertion, thereby preventing MSP and reducing consequences of MSP (e.g., sickness absence) among childcare workers.

Participatory ergonomics programs are commonly used as workplace interventions for prevention of MSP [6]. The involvement of the workers in the process is essential because it ensures relevance and that participants take responsibility for and get ownership of risk identification, solution development, and implementation of change [7], which are important for intervention effectiveness [8, 9]. The participatory ergonomics process is believed to encourage workers to be involved in optimizing their own work routines, consequently decreasing work-related risk factors [10] and thereby improving their health [11]. However, evidence on the effectiveness of participatory ergonomics for reducing MSP is incoherent [6, 12–14]. One reason for the lack of consistent findings may be related to the implementation process [15, 16]. A reason for poor implementation could be that most occupational interventions are considered sideline activities with limited relevance for the core work task of the workplace [17]. It has been emphasized that integrating the working environment and workplaces’ core work tasks are key factors for enhancing implementation and securing management support [17, 18]. Another reason may be related to difficulties in evaluating participatory interventions. Owing to the participatory approach, we do not know much about the actual content of a participatory ergonomic intervention, such as risk identification or solution development, which then becomes a black box [19]. Therefore, participatory ergonomics interventions need to focus on implementation factors specifically in the development of the intervention, but they also need to have a sound scientific evaluation design.

In the development of an intervention, it is important to focus on both effectiveness and feasibility. Effective interventions that are not feasible to be implemented are useless in practice, and the same applies to interventions that are feasible to be implemented but lack effectiveness [20, 21]. Feasibility and implementation of an intervention are therefore key factors to be considered during the development of an intervention [22]. A participatory approach in developing the intervention content is shown to predict engagement in intervention activities [9], and better implementation of the intervention is well documented to lead to greater effect [8]. Effective intervention development requires a linkage between a resource system (developers, such as researchers), an intermediate user system (implementers, such as occupational health and safety consultants or therapists), and an end user system (program participants, such as the workplace and the workers) [22]. Within health promotion research, interventions have been developed and implemented in a structured manner by use of, for instance, intervention mapping [22]. This process includes both knowledge obtained from the literature and involvement of key stakeholders to develop, implement, and evaluate an intervention [22]. Intervention mapping has proven useful in several occupational health studies, where it has been used to support the development of workplace interventions focusing on return to work [23, 24] and different health outcomes [25–27]. Therefore, intervention mapping will also be used to develop the intervention for the current study.

The aim of this study is to develop and implement a participatory ergonomic intervention and evaluate whether it is effective in reducing physical exertion and MSP (including pain-related work interference) among childcare workers through minimizing risk factors perceived by the workers themselves*.* The main aim of this paper is to describe the development, design, and evaluation of an intervention among Danish childcare workers. More specifically, the study has two main hypotheses that a participatory ergonomic intervention will:

1. Reduce physical exertion among childcare workers compared with usual practice

2. Reduce MSP and pain-related work interference among childcare workers compared with usual practice

## Methods/design

### Study design and participants

**Fig. 1:**
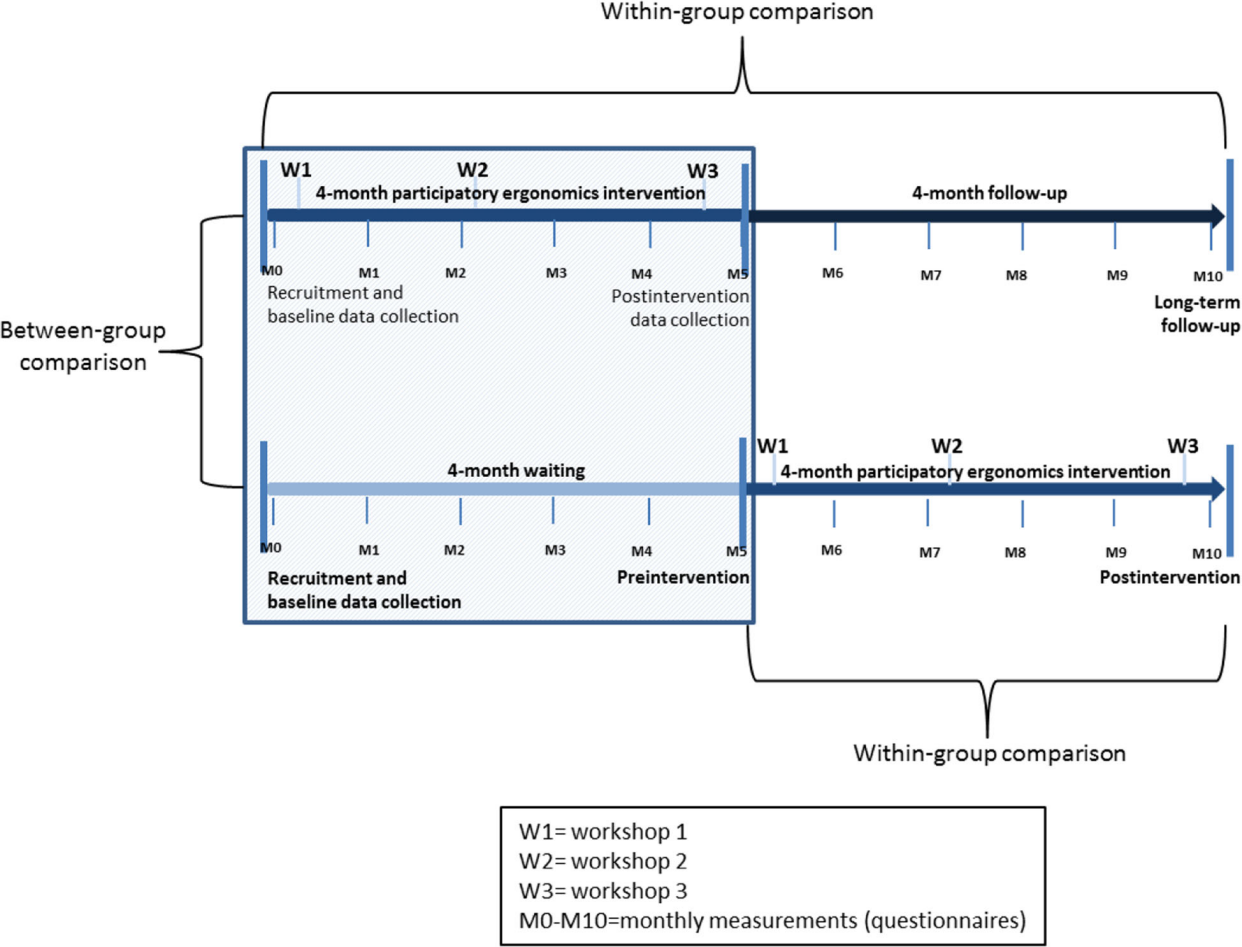
The design of the study and data collection points

In clinical intervention research, the randomized controlled trial (RCT) is considered the gold standard. However, in workplace settings, individual randomization cannot always be accommodated [28]. Therefore, this study uses a cluster-randomized design employing a wait-list control, with childcare institutions forming the clusters. Another concern in the workplace is that the introduction of control groups not receiving the intervention can hamper implementation due to impaired organizational commitment [29, 30]. Offering the intervention to the control group after the intervention has been implemented in the intervention group could be a solution for this. Therefore, childcare institutions are randomly assigned to two different arms (immediate/delayed intervention) (Fig. 1). All childcare workers from the randomized institutions were eligible for participation in the intervention, but participation in the evaluation of trial was voluntary. Before entering the trial, all childcare workers were asked to sign informed consent forms. The project began in the second half of 2017 with recruitment of participants, baseline measurements, and initial intervention meetings. This protocol conforms to the Consolidated Standards of Reporting Trials (CONSORT) guidelines [31], and future results of the study will be reported according to these guidelines.

### Study population

Childcare institutions in the Copenhagen Municipality are divided into five administrative divisions covering all public institutions as well as some private institutions (approximately 350 institutions in total). Each of the five divisions has its own manager. The project was presented to all five managers, all of whom were willing to offer participation to the institutions in their respective divisions. Within each division, the institutions are organized into a number of groups (range, six to nine groups) of institutions (range, three to eight institutions per group), each of which has an institution group manager. Information from the divisional managers was provided to the institution group managers, who then provided information to the institutions. Eligibility criteria for the institutions were childcare institutions for children aged 0–3 years and a minimum of nine employees (childcare workers). All childcare institutions in the Copenhagen Municipality fulfilling the eligibility criteria were invited to participate and give their response to the Work Environment Consultancy of Copenhagen (provides consultancy on all work environment issues in the Copenhagen Municipality and those responsible for delivering the intervention; *see* the “Intervention development” section below for further details). Thirty-three institutions responded positively to the invitation; one turned out to be too small (only six employees). We excluded three institutions because they had recently held an ergonomics course delivered by the Work Environment Consultancy of Copenhagen. Thus, there were 29 eligible institutions in total.

### Data protection, ethical approvals, and trial registration

The National Research Center for the Working Environment has an institutional agreement with the Danish Data Protection Agency about procedures to treat confidential data (journal number 2015-41-4232), such as by securing data on a protected drive with limited access and making all individual data pseudonymous. The Danish National Committee on Biomedical Research Ethics (the local ethics committee of Frederiksberg and Copenhagen) has evaluated a description of the study and concluded that, according to Danish law as defined in Committee Act § 2 and § 1, the intervention described should not be further reported to the local ethics committee (reference number 16048606). The study is registered in the ISRCTN Registry (ISRCTN10928313).

#### Randomization and blinding

All childcare institutions gave initial agreement to participate before we did the randomization. Because the intervention is group-based, and to avoid contamination between workers, cluster randomization was performed, with each childcare institution constituting a cluster. The randomization was balanced on institution size. Childcare institutions with 9–24 workers were stratified according to size to include an equal number of small (9–12 workers) and large (12+ workers) institutions. Within each size grouping, institutions were randomly allocated to either group 1 (early participatory ergonomics intervention) or group 2 (initial control group and late participatory ergonomics intervention group). The study was dimensioned to enroll approximately 200 workers (*see* the “Power calculation” section below). Because there were 29 institutions willing to participate with a total of approximately 400 workers, not all institutions could be offered an opportunity to participate in the study. In case some institutions dropped out before baseline data collection, we chose to randomize all 29 institutions so that we could replace that institution with another comparable institution. Thus, each institution was assigned a randomly drawn unique priority number between 1 and 100 and subsequently ranked within each group (InterventionSmall, ControlSmall, InterventionLarge, ControlLarge) with smaller number equaling higher priority. Finally, pairs were drawn until we reached the desired number of workers as demanded by the power calculation.

An independent data manager performed a computer-generated randomization using SAS for Windows statistical software (SAS Institute, Cary, NC, USA) developed by an independent statistician. Blinding of participants is not possible, owing to the nature of the trial. However, data collection will be performed using text messages, and persons collecting/handling data will be blinded to group allocation.

### Intervention

#### Intervention development

To ensure that the intervention are optimally tailored to the workplaces, the activities are developed using an intervention mapping approach [22]. The intervention mapping facilitates participation of all involved in the study. The development of the intervention activities is based on four key points:

1. *Effectiveness*: The activities should be effective, meaning that they should be theoretically sound and based on empirical findings from previous studies showing positive results.

2. *Feasibility*: The activities should be implemented at the workplace during working hours.

3. *Motivation*: Workers should find the activities appealing and relevant.

4. *Evaluation*: It should be possible to conduct a sound scientific evaluation, meaning that the activities follow a standardized protocol [22].

The first step of the intervention mapping concerns a needs assessment. The Work Environment Consultancy of Copenhagen had a significant rise in contacts from childcare institutions due to workers experiencing high physical work demands and MSP. This was confirmed in a nationally representative survey on health and work environment showing that childcare workers in Denmark report a high physical workload, high physical exertion during work, and high prevalence of MSP, as well as a high prevalence of sickness absence [5].

Before initiating the study, relevant scientific literature and previously conducted studies in this occupational group were used to identify interventions that are effective for reducing physical workload, physical exertion, and MSP. However, not many studies have been conducted for this occupational group [32]. We then searched the literature for intervention studies aiming to reduce physical exertion during work and MSP in other occupational groups, and we found that participatory ergonomics was a relevant intervention (*see* “Background” section above). The consultants from the Work Environment Consultancy of Copenhagen have many years of experience with childcare institutions, and they provided valuable information about preventive initiatives for physical workload and MSP that might work in practice. Overall, the intervention activities were codeveloped with the consultants combining information from the scientific literature with knowledge from practice.

Together with the consultants, we discussed the feasibility of implementing the activities in the childcare institutions. For optimal implementation of the activities, it was decided that the activities should primarily be conducted during existing staff meetings. In order to involve the participating workplaces and further tailor the intervention to childcare institutions, we conducted worksite visits to get insight into the working conditions and to better plan the implementation of the activities. We visited six different childcare institutions and conducted observations and brief interviews with the workers to get information about the physical workload and what they perceive as barriers to implementation of initiatives. Along with information from the literature, these data were used to fit the intervention content and implementation of the intervention into the existing strategies and to make the intervention feasible for the workplace.

**Fig. 2:**
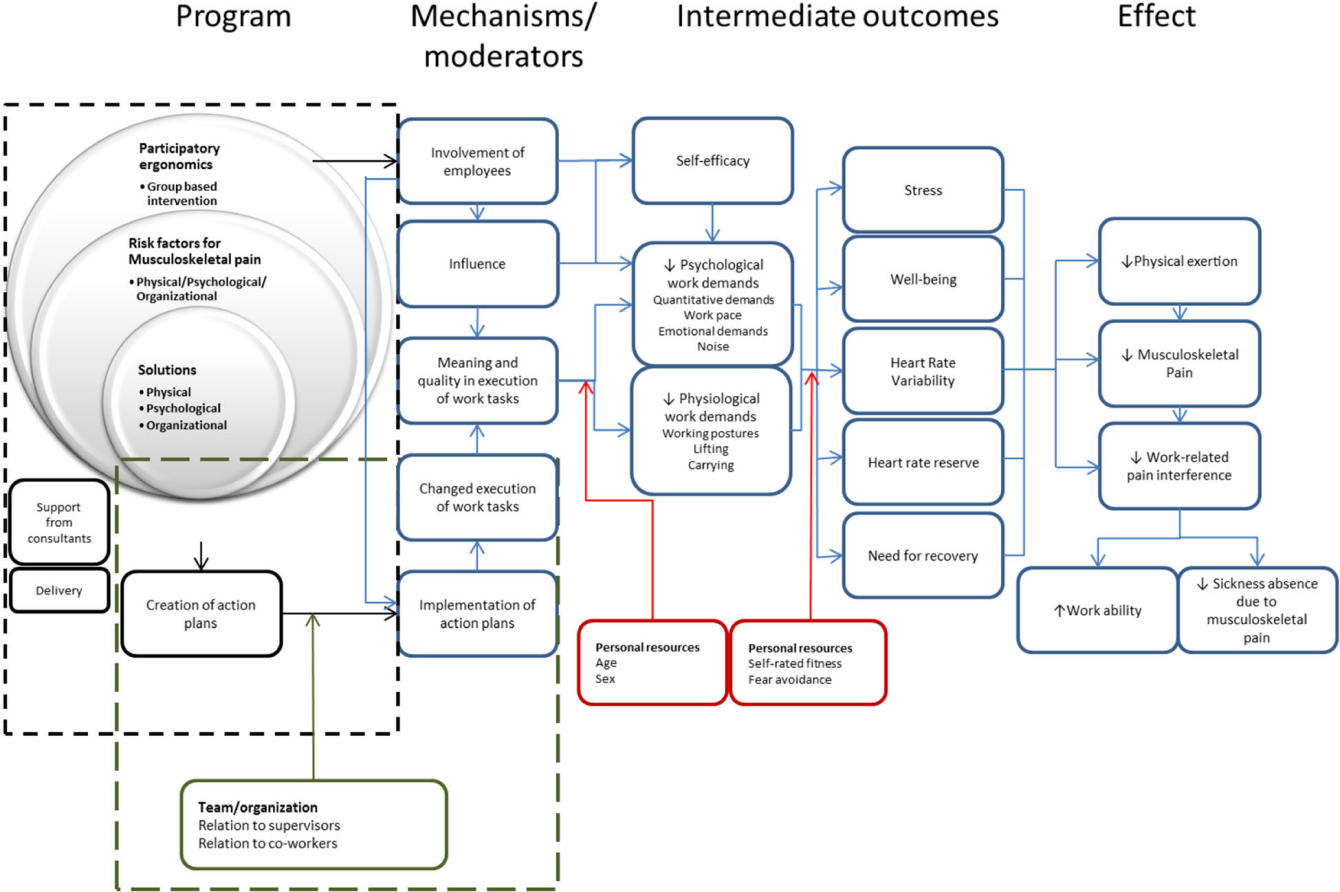
Program logic of the intervention. The program logic model of the study illustrates the intervention (the program) and the expected mechanisms and effects

Finally, a program logic was developed (Fig. 2). The program logic describes the mechanistic pathway from the intervention to the reduction in physical workload physical exertion during work and reduction in MSP among childcare workers. Moreover, the program logic also helps guide both the effect evaluation and process evaluation (*see* the “Process evaluation” section below for further details).

#### Delivery of the intervention

Ergonomic consultants from the Work Environment Consultancy of Copenhagen (occupational therapists and physiotherapists) were trained to carry out the intervention activities. In addition, a written intervention protocol describing all intervention activities was made. The working time spent on the activities by the workers will be financed by the workplace. The ergonomic consultants are not involved in the evaluation.

#### Intervention content: participatory ergonomics

**Fig. 3:**
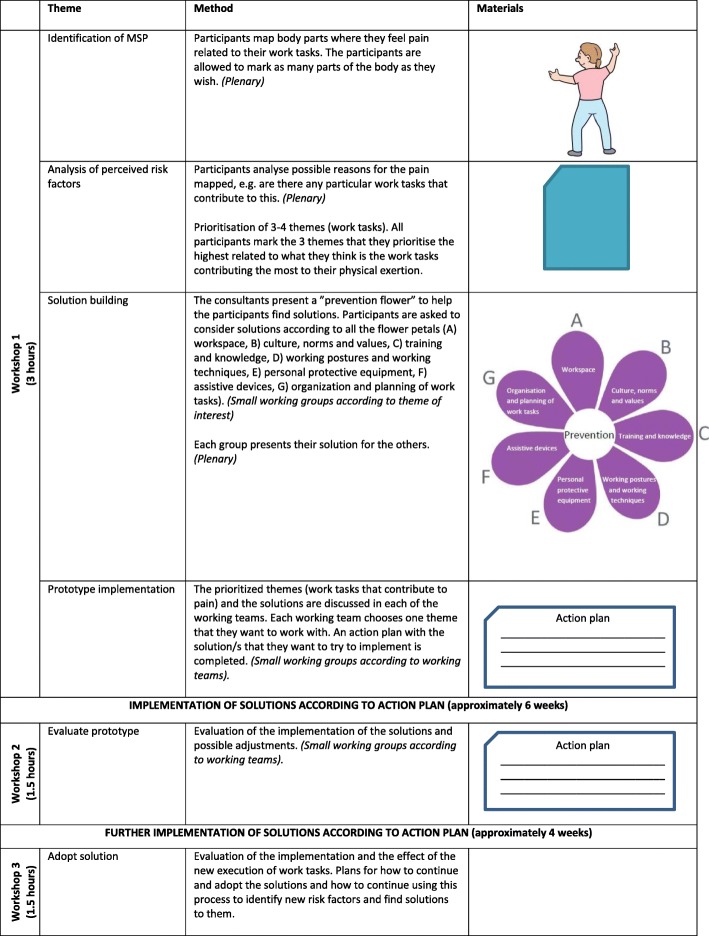
Overview of intervention activities and materials used in the intervention. The intervention is an organizational intervention, meaning that the activities are carried out in working teams. The intervention consists of three workshops covering different themes. Between the workshops, the working groups are expected to implement the suggested solutions according to the action plan. The methods vary between plenary discussions and smaller-group discussions in teams. A variety of materials are used throughout the intervention

Participatory ergonomics covers “the involvement of the workers in planning and controlling significant amount of their own work activities, with sufficient knowledge and power to influence both processes and outcomes to achieve desirable goals” [10]*.* The literature highlights participatory ergonomics as being not a unitary concept, but rather an umbrella term covering a broad range of ideas and practices [11]. In this study, inspiration from the framework suggested by Haines and colleagues [11] as well as the blueprint suggested by Wells and colleagues [33] was used in the development of the participatory ergonomics intervention. Therefore, the participatory ergonomic process follows 6 steps: (1) identification of risk factors, (2) analysis of risk factors, (3) solution building, (4) prototype implementation, (5) evaluation of prototype, and (6) adoption of a solution. These steps will be carried out in one workshop of 3 hours and two follow-up workshops of 1.5 hours (Fig. 3). A main feature of this participatory ergonomics intervention is the integration with the core work tasks as previously recommended for improving implementation [17].

All included childcare workers at the institutions will be involved in the participatory ergonomics, and the ergonomic consultant will guide the process. At the first workshop lasting 3 hours, the workers will identify work tasks that entail high physical workload that they perceive as risk factors for MSP and will analyze them. The results of this workshop should be three or four prioritized work tasks that should be (1) relevant (e.g., many workers perform the task, or the task is done many times during a working day) and (2) entail high physical workload or high physical activity. Further, the workers are asked to find solutions to the prioritized work tasks and make an action plan. They are again asked to prioritize the solutions according to (1) efficiency (i.e., can this solution reduce physical workload, physical exertion, and MSP?), (2) feasibility (i.e., is this solution likely to be implemented within the project period?), and (3) integration with the core work task (i.e., can this solution also improve the performance of the core work task?). After the workshops, the solutions should be implemented. At two follow-up meetings lasting 1.5 hours each, the implementation of the solutions will be evaluated, and possible adjustments will be made. If the workers find the implementation of a solution successful, the solution will be recommended for permanent adoption.

### Control

Those in the initial control group will continue their usual practice from baseline to the 4-month follow-up. Usual practice in this type of workplace is ergonomic consultancy and guidance or individual advice on pain management from therapists (physiotherapists or occupational therapists) employed in the municipality. Each workplace or worker can contact a therapist if they feel that they need it. It is not possible to collect information on an individual level with respect to use of a therapist, but we will have information on a workplace level whether they have contacted the therapists for ergonomic consultancy and guidance. This group will receive the intervention after the 4-month follow-up.

### Data collection

Data will be collected for all workers at three time points: baseline, 4-month follow-up, and 8-month follow-up (Fig. 1). For practical reasons, the baseline measurement took place after randomization but before the participatory ergonomics intervention. This was done because the workplaces need to plan their activities in advance, so the immediate intervention group needed information about startup to plan the workshops that were carried out as part of the intervention. The childcare workers were only told when their intervention started, with no mention of being in a control group or not. At baseline, an electronic questionnaire will be sent to all participants via a link in a text message, and they will be invited to participate in anthropometric measures as well as objective measures of physical activity and heart rate. Additionally, each worker will be observed during working hours.

Anthropometric measures will be taken at baseline during a half-hour session with trained clinical personnel (physiologists and physiotherapists). The participants will be asked to respond to electronic questionnaires throughout the project period, with short electronic questionnaires sent via a link in a short text message every 4 weeks and a longer electronic questionnaire sent after 4 months of follow-up and after 8 months of follow-up. Technical measurements of physical workload and physical activity will be performed for 3–5 days, and measurement of heart rate will be performed for 3–5 days at baseline as well as at 4-month follow-up (details are provided below). Trained clinical personnel (physiologists and physiotherapists) will mount the measurement devices. Observations will be carried out for 4 hours per worker, both at baseline and at 4-month follow-up, by personnel who have been trained according to a standardized protocol.

#### Questionnaires

The questionnaires contain standard and validated measures. They include sociodemographic information (i.e., sex, ethnicity, work-related factors [seniority, weekly working hours]), health and behavior (i.e., MSP) [34], medicine use, smoking, general health [35], self-efficacy [36], fear avoidance [37], well-being [38], self-rated physical capacity (aerobic fitness, muscle strength, balance, endurance, flexibility) [39], stress [40] and work environment factors (i.e., perceived physical exertion during work) [41], pain-related work interference [34], noise, psychosocial work environment measured by the Danish Psychosocial Questionnaire (new questionnaire that is currently in the process of being published), work ability [42], need for recovery [43], and sickness absence and presenteeism [44–46].

### Anthropometric measures, grip strength, and blood pressure

To assess the health of the participants at baseline, objective physical measures of body height (Seca 213; Seca GmbH, Hamburg, Germany), body weight, body fat percentage (BC-418 MA body composition analyzer; Tanita, Tokyo, Japan), body mass index (body weight [kg]/(body height [m^2^]), grip strength (Jamar NC70144; North Coast Medical, USA), and blood pressure (Omron M3 or Omron M6 Comfort; Omron Corporation, Kyoto, Japan) will be performed. Participants receive individual feedback on the results of the measurements.

### Physical activity

#### Body postures and movements

Technical measurements of physical activity type (e.g., walking, climbing stairs, running), postures (e.g., arms above shoulder height, bending of the back), body position (e.g., standing, sitting, kneeling, and lying), and steps will be performed using the validated Acti4 software [47, 48] using AX3 accelerometers (3-Axis Logging Accelerometer; Axivity Ltd., Newcastle upon Tyne, UK). Objective measures for twisting of the back will be performed by the Acti4 software and GT9X (GT9X Link; ActiGraph LLC, Pensacola, FL, USA) inertial measurement unit (IMU). Acti4 has been validated for estimation of physical activity types, postures, and movements in semistandardized settings and in free living [47–50].

The AX3 accelerometer provides measurements of linear accelerations in three dimensions with a dynamic range of ± 8 G, sampled with a precision of 13 bits. The AX3 accelerometers will be initialized for recording, and data will be downloaded using the manufacturer’s software (OMGUI Version 1.0.0.30; Axivity Ltd) at a sampling rate of 25 Hz. The GT9X provides nine-component motion sensing by measuring acceleration in three dimensions with a dynamic range of ± 16 G, angular velocity in three dimensions with a dynamic range of ± 2000 degrees/s, and magnetometer heading in three dimensions with a dynamic range of ± 4800 μT. The GT9X IMUs will be initialized for recording, and data will be downloaded using the manufacturer’s software (ActiLife version 6.13.3; Actigraph LLC) at a sampling rate of 100 Hz. The AX3 and GT9X will be mounted on the skin with adhesive tape (Hair-Set double-sided adhesive tape; 3M Company, Maplewood, MN, USA) and secured with transparent adhesive film (OPSITE FLEXIFIX; Smith & Nephew plc, London, UK).

Five AX3 accelerometers will be mounted for 4–5 days at the following positions:

1. The trunk, at one of two positions
  - The spine just below the processus spinosus at the level of T1-T2
  - On the midline of the flat part of the manubrium of the sternum
2. The dominant arm, laterally and 3 cm distal to the deltoid insertion
3. The right thigh at the most muscular part of the quadriceps femoris, midway on the line between the anterior inferior iliac spine and the top of the patella
4. Right and left calf, on the flat part of the soleus and gastrocnemius aponeurosis just distal to the lateral and medial heads of the gastrocnemius

Two GT9X IMUs will be mounted for 1 work day at the following positions:

1. Upper back, on the spine just below the processus spinosus at the level of T1-T2 (just distal to the AX3)
2. Lower back, on the spine at the level of L5-S1

The AX3 accelerometers and GT9X IMUs will be mounted with a spatial orientation of the accelerometer as described previously [47], with the *x*-axis being vertical and perpendicular to the skin surface with the positive *x*-axis pointing downward, the *y*-axis being horizontal and perpendicular to the skin surface with the positive *y*-axis pointing to the left, and the *z*-axis being horizontal and orthogonal to the skin surface with the positive *z*-axis pointing outward from the skin surface.

### Heart rate and heart rate variability measures

The measurements of heart rate and heart rate variability will be performed using Actiheart monitors (Actiheart; CamNtech, Cambridge, UK). The Actiheart is validated for measurement of heart rate and heart rate variability [51–53]. Actiheart measures raw electrocardiographic (ECG) signals with a sensitivity of 0.250 mV, which is electronically amplified by a factor of 900 (amplifier frequency response, 10–35 Hz). The resulting ECG signal is sampled at a frequency of 128 Hz, and each R-wave decaying edge is identified by using the Pan-Tompkins real-time QRS detection algorithm [54]. The Actiheart is configured to “short-term recording,” in which each interbeat interval (IBI) between consecutive detected R peaks in the QRS complex is stored for approximately 440,000 heartbeats. Before attachment, the skin will be shaved if necessary (Wilkinson Sword disposable hospital razor; Edgewell Personal Care, St. Louis, MO, USA) and gently rinsed with alcohol swabs (70% isopropyl alcohol). The Actiheart will be mounted with Ag/AgCl pregelled electrodes (Ambu BlueSensor VL-00-S/25; Ambu A/S, Ballerup, Denmark) at one of the two validated positions [55]:

1. The apex of sternum with a horizontal wire to the right at the level of the fifth and sixth intercostal space
2. The manubrium of the sternum with a horizontal wire to the right at the level of the second and third intercostal space

The Actihearts are initialized for recording, and data are downloaded using the manufacturer’s software (Actiheart 4, version 4.0.116; CamNtech). The Actiheart IBI recordings will be processed in the Acti4 software to derive heart rate (HR) and heart rate variability (HRV) features using robust period detection [47].

### Observational measures

Visual workplace observations will be carried out following a standardized manual for observation to capture physical activities we are unable to capture by objective measurements (e.g., lifting, carrying and physically supporting a child, as well as squatting and sitting on the floor). Additionally, observations will capture contextual information from situations (e.g., diaper change, clothes change) and barriers to work (e.g., disturbances). Visual observations of large body postures and work activities are shown to have reasonable reliability among trained observers [56]. The observations will be carried out using a modified TRAC (task recording and analysis on computer)/portable ergonomic observation approach [57, 58]. In short, continuous observation will take place using a handheld computer (GT-P3100 or SM-T280; Samsung, Suwon, South Korea) with Pocket Observer software (Pocket Observer version 3.3.46; Noldus Information Technology, Wageningen, The Netherlands) to record start-stop or occurrence of observed items. The Pocket Observer recordings will be processed using The Observer XT software (The Observer XT version 14; Noldus Information Technology) to derive duration and frequency of observed items.

### Outcomes

#### Primary outcome measures

The primary outcomes of this study are (1) self-rated physical exertion measured on a Likert scale (0–10) [41] every 4 weeks by questionnaire from baseline to 4 months and (2) MSP (duration, intensity, and number of pain regions and pain-related work interference) measured every 4 weeks by questionnaire from baseline to 4 months.

#### Secondary outcome measures

**Fig. 4:**
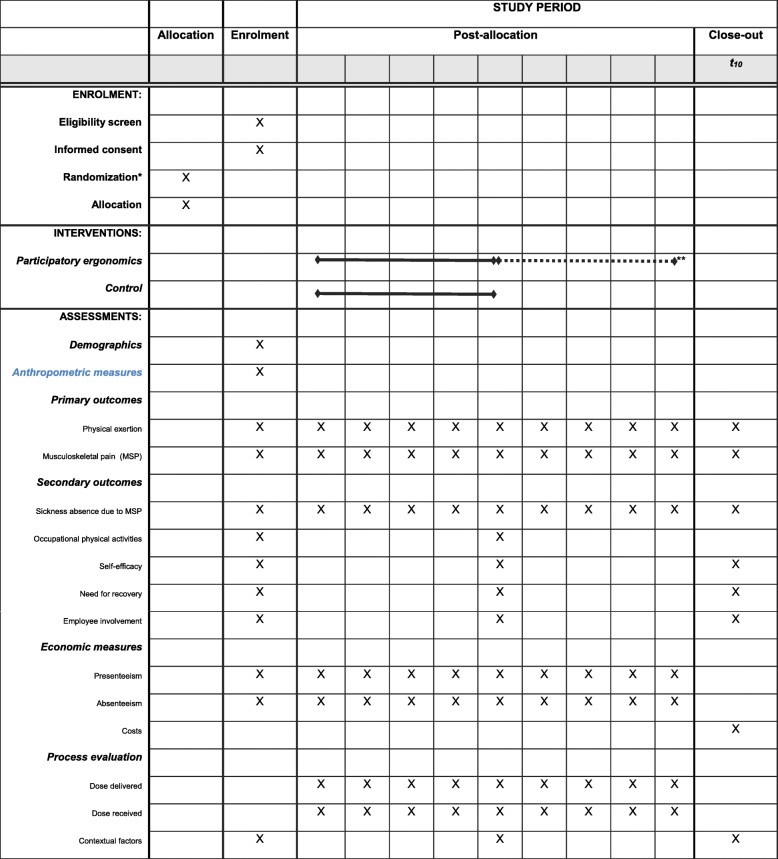
Standard Protocol Items: Recommendation for Interventional Trials (SPIRIT) schedule of enrollment, interventions, and assessments [59]. *The randomization was carried out at workplace level before baseline measurements. However, the childcare workers were only told when their intervention started, with no mention of being in a control group or not. **The study uses a wait-list design, so that the control group receives the intervention after the immediate intervention group

Secondary outcomes of this study are (1) self-reported sickness absence due to MSP (days) measured by questionnaire every 4 weeks from baseline to 4 months, (2) objectively measured occupational physical activities by accelerometers and heart rate monitor at baseline and at 4 months, (3) self-efficacy measured by questionnaire at baseline and at 4 months, (4) need for recovery measured by questionnaire at baseline and at 4 months, and (5) employee involvement measured by questionnaire at baseline and at 4 months. Figure 4 shows the Standard Protocol Items: Recommendation for Interventional Trials (Additional file 1: SPIRIT) [59] schedule of enrollment, interventions, and assessments.

#### Power calculation

For the power calculation, we used the calculation for a clustered parallel group with before-and-after measures to calculate the design effect [60]. Statistical power analysis was performed for primary outcome measures. The scale for self-rated physical exertion (0–10) has a mean of 4.09 and an SD of 2.41 in a representative sample of childcare workers in Denmark (*N* = 1137) [5]. The intracluster correlation coefficient (ICC) was calculated on the basis of a previous participatory ergonomics intervention among eldercare workers [61]. With a power of 0.80, an alpha of 0.05, a fixed cluster size of 12, and an estimated ICC of 0.005, we need 192 participants (96 per group) corresponding to approximately 16 clusters to statistically demonstrate a relevant effect in physical exertion of 1 point [60]. We also tried to vary the cluster size between 9 and 20 and check for differences in changing the values of ICC, which did not change the anticipated power very much. Because this is an organizational intervention, we expect only a small number of dropouts. In addition, the intervention period is quite short. We therefore expect to include 10% more than needed according to our power calculations, so that at baseline we will include approximately 210 participants. Additional power calculation for MSP showed that the numbers needed are within the same range as calculated for physical exertion.

### Economic measures

#### Health-related production loss

Presenteeism (reduced performance while being present at work) and absenteeism (hours and days missed from work due to sickness absence) will be measured by questionnaire every 4 weeks [44, 45]. In addition, sickness absence data (absenteeism) will be retrospectively collected from company records after the last follow-up measurement.

#### Cost measures

Intervention costs include the costs for workshops (i.e., costs for the consultant [including preparation], time spent on the workshop by the participants, and materials), and costs for meetings with the workplace in preparation for the project, as well as costs for fruit and snacks offered at these meetings and printed materials. Consultants’ and participants’ costs will be valued on the basis of their salary. Other costs will be valued using invoices. Workplace productivity losses (i.e., presenteeism and absenteeism) will also be valued using salaries of the participants.

### Economic evaluation

The economic evaluation aims to determine the cost-effectiveness of the intervention compared with usual care from the employer’s perspective. In addition, a cost-benefit analysis will be performed from the employer’s perspective. Analyses will be performed according to the intention-to-treat principle with multiple imputation of missing data [62, 63]. Sensitivity analyses will be done to assess the robustness of the results. The total employer’s costs of the intervention will be estimated and compared between the intervention and control groups. The 95% CIs will be estimated using approximate bootstrap CIs [63]. For the cost-effectiveness analysis, incremental cost-effectiveness ratios will be calculated by dividing the mean difference in costs between both groups by the difference in effects on the primary outcome measures self-rated physical exertion and MSP. Cost-effectiveness planes will be graphically presented [62]. In addition, a cost-benefit analysis will be performed in which the incremental intervention costs will be compared with the incremental productivity-related costs. This will be expressed as return on investment as well as benefit-to-cost ratio.

### Process evaluation

The interpretation of interventions implemented in workplaces can be a challenge. A mere effect evaluation explains only a fraction of the causal assumptions in the program logic [8, 64], and the effect evaluation itself risks rejecting the hypothesis underlying the program theory due to implementation failure (type III error). Thus, both the effects and the implementation (processes) need to be evaluated. Analyzing the effects and processes of an intervention requires a comprehensive evaluation [65].

A process evaluation will be performed, inspired by the framework by Steckler and Linnan [20] to gain insight into the extent to which the intervention is implemented as intended [65]. The implementation will be measured through dose delivered (the amount of intervention components delivered by the consultants) and dose received (employees’ participation in the activities) [20]. The delivery will be measured by asking the consultants to what extent they have followed the specific intervention activities in accordance with the defined criteria written in the intervention protocol (they will fill out a questionnaire after each activity). To check the quality of the consultants’ responses to the questionnaires, observations of the workshops by an independent observer will be conducted (by filling out a similar questionnaire). The dose will be measured by participation percentage and by questionnaires to the participants, after the intervention, asking about their appraisal of the intervention [20]. Moreover, information about the context will be collected through questionnaires from the managers of each of the childcare institutions (e.g., whether there have been any great organizational changes throughout the project period and whether there have been any concurrent activities in both the intervention and control groups that might influence both the implementation and the effect of the study).

### Statistical analyses

Baseline characteristics will be described by questionnaires and the anthropometric measures. Analyses regarding the effectiveness of the primary outcomes and secondary outcomes will be performed after 4 months of intervention with multilevel analyses. Multilevel analyses take clustering of observations of workers within the same team into account, as well as repeated measurements within one participant [66]. All analyses will be performed according to the intention-to-treat principle, including all eligible randomized participants without imputations because mixed models inherently account for missing values [67]. Both the immediate intervention group and the delayed intervention group will be followed with questionnaires and objective measurements after the delayed intervention group has received the intervention. It is expected that during this time, the immediate intervention group will continue with the intervention activities. This allows for longer follow-up for the immediate intervention group and the possibility to do a secondary analysis of long-term follow-up. However, due to the design of the study (two-arm cluster-randomized design employing a wait-list control), no untreated control group exists at the measurement after 8 months, and follow-up effects will thus be uncontrolled and modeled using data from intervention participants only (Fig. 1).

## Discussion

This paper describes the study protocol of the participatory ergonomic intervention among childcare workers called “Improving work for the body” (the TOY-project). It is hypothesized that successful implementation of the TOY-project will lead to a reduction of physical exertion and MSP after 4 months.

### Strengths and limitations of the study

The cluster-randomized controlled trial design is a methodological strength because it minimizes the risk of contamination between the intervention and control groups and reduces the risk of bias. The systematic intervention mapping approach is a strong feature of the study. The experience and information obtained in the process of tailoring and developing the intervention will be captured and, hopefully, benefit both the present and future studies. Another strength is the monthly monitoring of the outcomes. Moreover, as recommended by Takala and colleagues, we will use multiple tools to capture different aspects of physical workload [56]; such as objective measurements, observations, and questionnaires. This will also allow a rigorous description of the physical workload and physical activity at work in this occupational group. By also incorporating a process evaluation, we will gain insight into the implementation process within the intervention teams and into potential parallel activities in control teams. Another strength is that consultants will deliver the intervention, and these consultants are not involved in the evaluation. The results should be generalizable to similar workplaces, and we will conduct an economic evaluation to evaluate the cost from an employer’s perspective.

Because this is an organizational intervention, it does not focus on individual workers. Thus, individual randomization is not feasible. Our trial lacks allocation concealment and will be at risk of selection biases. However, we will check and report any evidence of selection bias by comparison of the proportion that participated at each institution and compare characteristics (by using information from the payroll) of participants and nonparticipants. Moreover, due to the interventional trial design, participants cannot be blinded to group allocation. However, all participating childcare workers would receive the intervention and are only told when their intervention is intended to start with no mention of being in a control group. This minimizes the potential selection bias. However, outcome assessors and data analysts will be blinded to group allocation. A possible limitation is the short follow-up duration, because the effect of workplace changes may take longer time to set in (4 months). In addition, the use of a wait-list design could imply a risk of implementing a noneffective intervention for the control arm, which will have cost and resource implications. Our experience with such interventions is that the process, no matter the statistical effect, will benefit the workers, and the workplaces need to plan these interventions a long time ahead and promise the employees that they will receive the intervention.

### Impact of the results

This intervention may benefit employees as well as employers. If the intervention proves to be effective, the childcare workers will benefit from an improved health and working environment. These positive effects may potentially contribute to reduce sickness absence and thereby be beneficial for society as well. Surprisingly little research has been conducted for childcare workers [32]. This study will contribute to closing the research gap for this occupational group. Results are expected in 2018–2019.

### Trial status

The study was opened to recruitment in August 2017. Recruitment ended in November 2017. The intervention ended in July 2018. The duration of the study period will be 3 years and finish in December 2019.

## Additional file

Additional file 1: SPIRIT 2013 checklist: Recommended items to address in a clinical trial protocol and related documents. (DOC 121 kb)

## Abbreviations

CONSORT: Consolidated Standards of Reporting Trials
ECG: Electrocardiographic
IBI: Interbeat interval
ICC: Intracluster correlation coefficient
IMU: Inertial measurement unit
MSP: Musculoskeletal pain
RCT: Randomized controlled trial
SPIRIT: Standard Protocol Items: Recommendations for Interventional Trials
